# Exploring the Proteomic Landscape of Cochlear Implant Trauma: An iTRAQ-Based Quantitative Analysis Utilizing an Ex Vivo Model

**DOI:** 10.3390/jcm14145115

**Published:** 2025-07-18

**Authors:** Jake Langlie, Rahul Mittal, David H. Elisha, Jaimee Cooper, Hannah Marwede, Julian Purrinos, Maria-Pia Tuset, Keelin McKenna, Max Zalta, Jeenu Mittal, Adrien A. Eshraghi

**Affiliations:** 1 Hearing Research and Cochlear Implant Laboratory, Department of Otolaryngology, University of Miami Miller School of Medicine, Miami, FL 33136, USA; jal141@med.miami.edu (J.L.); r.mittal11@med.miami.edu (R.M.); dxe421@miami.edu (D.H.E.); hxm352@med.miami.edu (H.M.); jxp2025@med.miami.edu (J.P.); kmcke018@med.fiu.edu (K.M.); 77mrzalta@gmail.com (M.Z.); j.mittal@med.miami.edu (J.M.); 2 Faculty of Medicine, Tel Aviv University, Tel Aviv 6997801, Israel; 3 School of Medicine, New York Medical College, Valhalla, NY 10595, USA; 4 Department of Neurological Surgery, University of Miami Miller School of Medicine, Miami, FL 33136, USA; 5 Department of Biomedical Engineering, University of Miami, Coral Gables, Miami, FL 33146, USA

**Keywords:** cochlear implantation, electrode insertion trauma, iTRAQ proteomics, organ of Corti, residual hearing preservation, molecular pathways

## Abstract

**Background:** Cochlear implantation is widely used to provide auditory rehabilitation to individuals with severe-to-profound sensorineural hearing loss. However, electrode insertion during cochlear implantation leads to inner ear trauma, damage to sensory structures, and consequently, loss of residual hearing. There is very limited information regarding the target proteins involved in electrode insertion trauma (EIT) following cochlear implantation. **Methods:** The aim of our study was to identify target proteins and host molecular pathways involved in cochlear damage following EIT utilizing the iTRAQ™ (isobaric tags for relative and absolute quantification) technique using our ex vivo model. The organ of Corti (OC) explants were dissected from postnatal day 3 rats and subjected to EIT or left untreated (control). The proteins were extracted, labelled, and subjected to ultra-high performance liquid chromatography–tandem mass spectrometry. **Results:** We identified distinct molecular pathways involved in EIT-induced cochlear damage. Confocal microscopy confirmed the expression of these identified proteins in OC explants subjected to EIT. By separating the apical, middle, and basal cochlear turns, we deciphered a topographic array of host molecular pathways that extend from the base to the apex of the cochlea, which are activated post-trauma following cochlear implantation. **Conclusions:** The identification of target proteins involved in cochlear damage will provide novel therapeutic targets for the development of effective treatment modalities for the preservation of residual hearing in implanted individuals.

## 1. Introduction

Cochlear implantation (CI) is a highly effective treatment for auditory rehabilitation in individuals with severe-to-profound sensorineural hearing loss (SNHL), offering significant improvements in hearing, social engagement, and overall quality of life. The CI bypasses damaged or absent cochlear structures, directly stimulating auditory neurons through electrodes inserted into the cochlea [1]. The clinical benefits of CI have been well-documented across diverse etiologies, including genetic disorders such as Alport syndrome and Usher syndrome [2,3], non-syndromic genetic causes like *GJB2* mutations [4], and acquired environmental factors such as ototoxicity or traumatic injury [5,6,7,8,9].

Historically, CI was reserved primarily for patients with minimal to no residual hearing. However, current indications increasingly encompass patients who retain significant residual hearing. Consequently, the preservation of residual hearing has emerged as a critical clinical outcome. Successful residual hearing preservation (HP) also facilitates electroacoustic stimulation, where acoustic and electrical inputs combine to yield richer, more natural sound perception, notably enhancing speech comprehension in noisy environments [10]. This bimodal auditory strategy often results in superior speech recognition compared to electrical stimulation alone. HP also supports smoother patient adaptation to CI, contributes to better music appreciation by preserving tonal quality, and improves spatial hearing by providing binaural auditory cues [11]. Furthermore, intact residual hearing and preserved cochlear structure improve outcomes in reimplantation procedures and may maintain patient eligibility for future regenerative therapies, such as hair cell regeneration [12,13,14,15,16]. Psychologically, preserved hearing confers greater emotional stability, reduced anxiety, improved self-confidence, and enhanced environmental awareness, particularly when the external processor is turned off [12].

Despite notable advancements in surgical techniques and electrode designs aimed at reducing intracochlear trauma, functional hearing preservation is not reliably achieved. One significant challenge is the perception of electrode insertion trauma (EIT), occurring during surgical electrode placement. This trauma can range from mild-to-moderate disruption of cochlear structures (grades 1–2) to severe damage such as scala vestibuli translocation or fracture of the osseous spiral lamina (grades 3–4), leading to inflammation, apoptosis, and progressive degradation of residual auditory function [10,11,12,17,18]. Consequently, reducing EIT is essential for improving hearing preservation outcomes and long-term CI success [19,20,21].

However, the specific molecular pathways and target proteins involved in cochlear trauma following implantation remain largely unexplored, across the different cochlear turns (apical, middle, and basal). Identifying these molecular responses could significantly advance the development of otoprotective strategies to mitigate cochlear damage and preserve residual hearing [22].

Advances in proteomics provide powerful and innovative approaches to explore these complex molecular mechanisms. Unlike genomic analyses, proteomics techniques reflect real-time cellular dynamics by capturing protein expression, post-translational modifications, and intricate protein–protein interactions [23,24,25]. These methods can characterize molecular networks, identify critical signaling pathways, and clarify the composition and activity of proteins within specific cellular compartments. This information is crucial for understanding cochlear responses to trauma and guiding therapeutic intervention [26,27,28,29,30,31].

Among these emerging technologies, iTRAQ™ (isobaric tags for relative and absolute quantification), a quantitative proteomic technique employing liquid chromatography–tandem mass spectrometry, stands out for its precision and versatility in analyzing protein dynamics across multiple biological samples [32,33,34,35,36,37,38,39]. Initially developed for yeast protein expression studies [40,41], iTRAQ™ is now broadly utilized to uncover the molecular underpinnings of various human diseases, such as hepatocellular carcinoma, diabetic nephropathy, and dermatological conditions [42,43,44,45,46,47]. Its quantitative accuracy makes it particularly suited for investigating complex pathological processes, including the molecular events associated with cochlear damage following electrode insertion trauma [48,49,50,51,52].

The objective of this study was to identify host molecular pathways and target proteins implicated in cochlear damage after EIT using the iTRAQ™ proteomic approach. Additionally, we aimed to confirm the localization and expression of these identified proteins within the apical, middle, and basal turns of the cochlea through confocal microscopy. Achieving a deeper understanding of these molecular mechanisms has significant clinical potential, paving the way for targeted otoprotective interventions that could substantially improve residual hearing preservation and enhance clinical outcomes for cochlear implant recipients.

## 2. Methods

**Figure 1:**
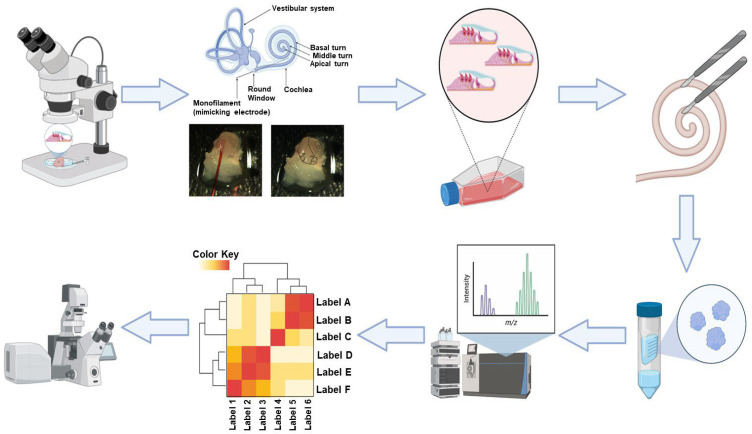
Graphical Representation of the Extraction and Processing of the Organ of Corti. A schematic overview of the experimental workflow, showing the dissection, protein extraction, labeling using iTRAQ™, separation, and analysis of proteins from the organ of Corti explants subjected to electrode insertion trauma (EIT) or untreated controls.

This animal study was approved by the Animal Care and Use Committee of the University of Miami and fully complies with the NIH guidelines for the care and use of laboratory animals. A graphical representation of the extraction and processing of the organ of Corti samples is shown in Figure 1.

### 2.1. Organ of Corti (OC) Dissections

OCs were dissected from three-day-old (P3) Sprague-Dawley laboratory rats (Charles River Laboratories, Inc., Wilmington, MA, USA) [53]. For EIT, a custom-designed electrode was introduced in the cochlea through the small 0.35 mm diameter cochleostomy located next to the round window niche, in order to achieve a high angle and depth of insertion into the scala tympani, which varied between 110 and 150 degrees as described in detail in previous studies. All cochleae were then incubated for 10 min in phosphate saline buffer (PBS) followed by excision of OC explants. OC explants were placed in serum-free media consisting of Dulbecco’s modified Eagle’s medium (DMEM) supplemented with glucose (6 g/L), N-1 supplement (1%), and penicillin G (500 U/mL). Explants were cultured at 37 °C in a 95% humidified atmosphere and 5% CO_2._ Explants were either subjected to EIT or left untreated (control). A total of 72 animals were used in this study, with equal distribution across treatment (EIT) and control groups (Supplementary Table S1). Each experimental condition was replicated in triplicate across at least three independent litters to account for biological variability. Untreated control explants were maintained under identical culture conditions as the EIT group but without electrode insertion. Each group (EIT and control) included explants from the apical, middle, and basal cochlear turns, processed in parallel.

### 2.2. Proteomic Analysis

#### 2.2.1. Protein Extraction and Quality Control

Following culture and treatment, OC explants were harvested, and total protein was extracted using a lysis buffer containing 8 M urea, 4% CHAPS, and protease inhibitor cocktail. Samples were sonicated on ice and centrifuged at 14,000× *g* for 15 min at 4 °C [54]. The supernatant containing soluble proteins was collected, and protein concentration was determined using the Bradford assay (Bio-Rad Laboratories, Hercules, CA, USA). Protein quality was assessed by SDS-PAGE prior to digestion and iTRAQ labeling.

#### 2.2.2. Trypsin Digestion and iTRAQ™ Labeling

Equal amounts of protein (100 μg) from each sample group (apical, middle, and basal cochlear turns post-EIT, and controls) were dried in a centrifugal vacuum concentrator to a final volume of <10 μL [54]. Samples were reconstituted in 30 μL of dissolution buffer (0.5 M triethylammonium bicarbonate, pH 8.5), followed by addition of 1 μL of denaturant solution (2% SDS). Reduction was carried out with 2 μL of freshly prepared 110 mM tris(2-carboxyethyl)phosphine (TCEP), incubated at 60 °C for 1 h. Subsequently, 1 μL of 84 mM iodoacetamide (in 100 mM ammonium bicarbonate) was added for alkylation in the dark at room temperature for 30 min. Digestion was performed by adding 10 μL of sequencing-grade modified trypsin (0.1 μg/μL; Promega, Madison, WI, USA), followed by incubation at 37 °C for 3 h [54]. An additional 1 μL of trypsin was added, and digestion was continued overnight (12–16 h) at 37 °C. After digestion, samples were labeled using the 4-plex iTRAQ Reagent Multiplex Kit (AB Sciex, Framingham, MA, USA) according to the manufacturer’s instructions. Labeled peptides were pooled and dried using a vacuum concentrator.

#### 2.2.3. Protein Identification and Quantification

Pooled iTRAQ™-labeled peptides were reconstituted in 98% Solvent A (0.1% formic acid in water) and 2% Solvent B (0.1% formic acid in acetonitrile), and loaded onto a C18 trap column (2 cm × 100 μm, 5 μm particle size) connected in-line with an analytical C18 column (25 cm × 75 μm, 2 μm particle size) using an EASY-nLC 1000 system (Thermo Fisher Scientific, Waltham, MA, USA) [54]. Peptides were separated with a linear gradient from 5% to 35% Solvent B over 120 min at a flow rate of 300 nL/min.

MS analysis was conducted on a Q Exactive Hybrid Quadrupole-Orbitrap Mass Spectrometer (Thermo Fisher Scientific) operating in data-dependent acquisition (DDA) mode. Full MS scans were acquired with an AGC target of 1 × 10^6^, followed by HCD fragmentation of the top 20 precursor ions with an AGC target of 2 × 10^5^, isolation window of 1.3 m/z, normalized collision energy of 28 eV, and resolution set to 17,500.

Proteins were identified and quantified from LC-MS/MS spectral data using Proteome Discoverer 2.2 software (Thermo Fisher Scientific, Waltham, MA, USA). Raw files were searched against *Rattus norvegicus* (rat) entries in the Uniprot sequence database using the Sequest HT search engine. Relative protein quantitation was based on the intensity of iTRAQ reporter ions released during MS/MS fragmentation [54].

### 2.3. Confocal Microscopy

OC explants were fixed in 4% paraformaldehyde, washed in PBS, and permeabilized with 0.2% Triton X-100. Non-specific binding was blocked with 5% normal goat serum for 1 h at room temperature. Samples were incubated overnight at 4 °C with primary antibodies against STK24 (Novus Biologicals, Centennial, CO, USA). Following PBS washes, samples were incubated with Alexa Fluor-conjugated secondary antibodies (1:500, Thermo Fisher Scientific, Waltham, MA, USA) for 1 h at room temperature. Hair cells were counterstained with FITC-phalloidin and nuclei with DAPI. Stained samples were mounted with antifade medium and imaged using a Zeiss confocal microscope. Images were analyzed using ImageJ software (version 1.53) for quantification of fluorescence intensity and hair cell viability. Antibodies and reagents used include STK24 primary antibody (Novus Biologicals, Centennial, CO, USA, catalog #NBP1-87833, 1:200), Alexa Fluor 568 goat anti-rabbit secondary antibody (Thermo Fisher, Waltham, MA, USA catalog #A-11036, 1:500), FITC-Phalloidin (Thermo Fisher, Waltham, MA, USA, catalog #A12379), and DAPI (Sigma Aldrich, St. Louis, MO, USA, catalog # D9542). All incubations were conducted in light-protected humidified chambers.

### 2.4. Statistical Analysis

Statistical comparisons between groups were performed using one-way ANOVA followed by Tukey’s post-hoc test for multiple comparisons. For pairwise comparisons, unpaired two-tailed Student’s *t*-tests were used. A *p*-value < 0.05 was considered statistically significant. For proteomic data, one-way ANOVA was used for group comparisons, followed by correction for multiple testing using both the Benjamini-Hochberg false discovery rate (FDR) method and the Bonferroni correction. Proteins with an adjusted *p*-value < 0.05 under either correction were considered significant, with FDR applied for broad discovery and Bonferroni used to identify high-confidence targets. GraphPad Prism 9.0 (GraphPad Software, San Diego, CA, USA) was used for all statistical analyses and data visualization.

## 3. Results

### 3.1. Gradient Loss of Sensory Cells from Apical to Basal Cochlear Turn in Response to EIT

**Figure 2:**
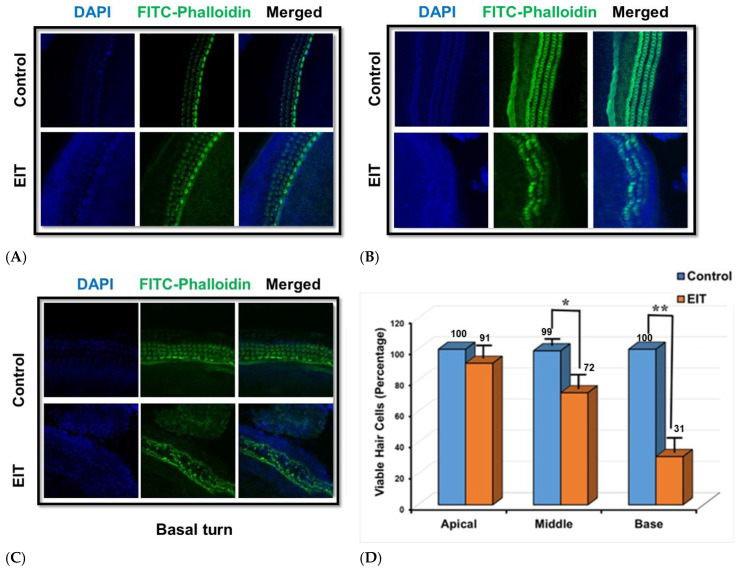
Ex vivo Model of Cochlear Implant Trauma. Confocal microscopy images showing the gradient loss of sensory hair cells in apical (**A**), middle (**B**), and basal (**C**) cochlear turns of the organ of Corti explants subjected to EIT. The images depict significant hair cell damage, particularly in the basal turn near the site of electrode insertion, compared to untreated controls. (**D**) The quantification of hair cell viability confirmed this data, showing significant reductions in hair cell viability in the middle and basal turns compared to the control group. * *p* < 0.01 or ** *p* < 0.001. DAPI-4′,6-diamidino-2-phenylindole; EIT-electrode insertion trauma. DAPI (blue) stains nuclei; FITC-Phalloidin (green) labels actin filaments in hair cells.

To confirm that our ex vivo model mimics EIT following cochlear implantation, we extracted OC samples and performed immunohistochemistry to determine the integrity of hair cells following trauma. We observed a sequential decrease in viable hair cells from the apical to middle to basal turn in OC explants subjected to EIT compared to the control samples. This is displayed in Figure 2A–C. The quantification of viable hair cells was confirmed using confocal microscopy showing significant loss of HCs from the basal to apical turn (Figure 2D). We observed that 93% of HCs survive in the apical turn. There was no statistically significant difference in viable HCs between the EIT and control samples in the apical turn. On the other hand, there was a significant decrease in viable HCs in the middle turn in OC subjected to EIT compared to the control samples (*p* < 0.01) (Figure 2D). The most significant decrease in viable HCs was observed in the basal turn where the electrode was inserted compared to the control samples.

### 3.2. Identification of Target Proteins Involved in EIT-Induced Sensory Cell Damage

Using iTRAQ™ technology, several novel upregulated proteins were identified in response to EIT in OC explants. Specifically, we analyzed proteins that were upregulated in the basal and middle cochlear turns, regions directly affected by electrode insertion trauma, but which showed minimal upregulation or only basal expression in the apical turn, which remained unaffected by the electrode. This spatial expression pattern allowed us to identify proteins potentially associated with EIT.

**Figure 3:**
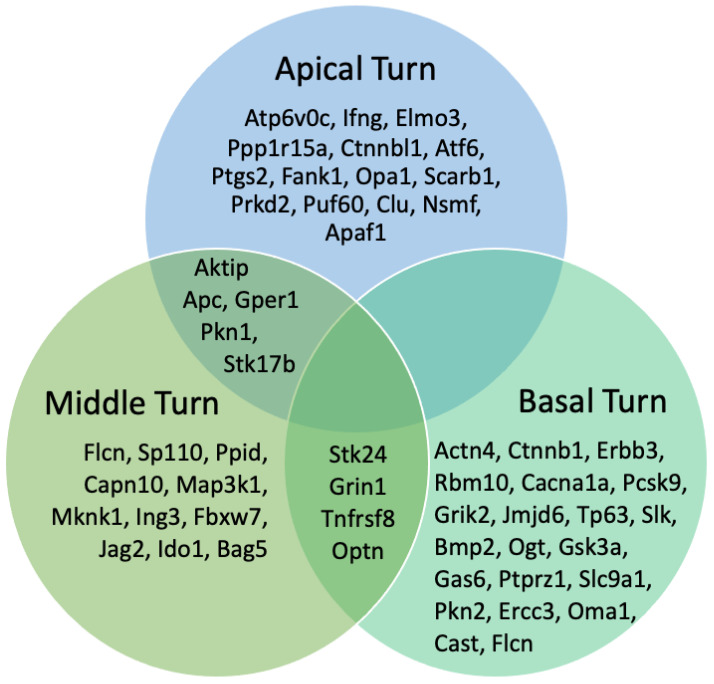
Venn Diagram of Top Upregulated Proteins Stratified by Cochlear Turn. Highly upregulated proteins in response to cochlear implant trauma are stratified based on topographic distribution. The proteins are separated by the apical, middle, and basal turns, with the proteins upregulated in multiple turns highlighted. Of specific interest, the apoptotic proteins Stk24, Grin1, Tnfrsf8, and Optn demonstrate upregulation in the middle and basal turns, coinciding with the sites of cochlear implant trauma, but do not demonstrate upregulation in the apical turn.

Four proteins were found to have high expression in the middle and basal turns, coinciding with the area of EIT, including STK24, GRIN1, TNFRSF8, and OPTN. Serine/threonine-protein kinase 24 (STK24), a novel marker identified in this study, phosphorylates both serine and threonine residues and promotes apoptosis in response to stress stimuli and caspase activation. The topographic expression of upregulated proteins in response to EIT is shown in Figure 3. The 20 highest upregulated proteins throughout the apical, middle, and basal turns are displayed in Table 1.

### 3.3. Confocal Microscopy

**Figure 4:**
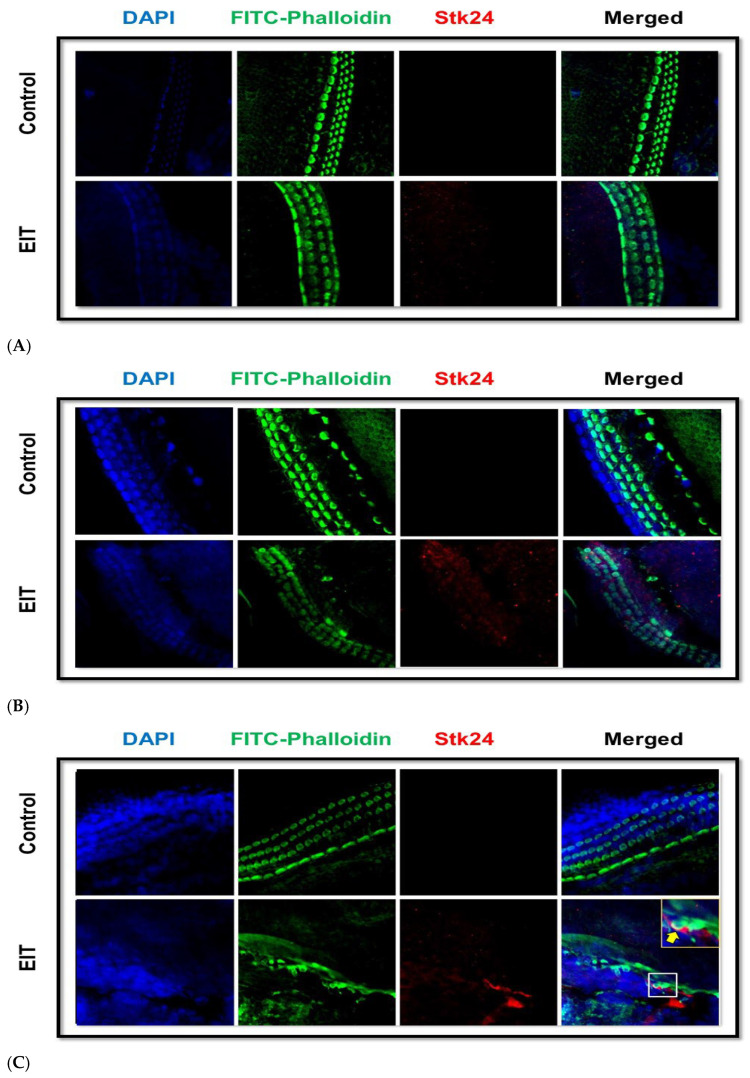
Stk24 Immunostaining. The apical (**A**), middle (**B**), and basal (**C**) turns of the organ of Corti explants from neonatal rat pups were subjected to immunostaining using DAPI (nuclear staining), FITC-Phalloidin (hair cell staining), and Stk24 (specific apoptotic protein). The insert shows Stk24 expression on and around the hair cells (HCs).

To confirm the findings of the iTRAQ analysis, the OC samples were subjected to STK24 immunostaining. In agreement with our iTRAQ results, we observed intense immunostaining of STK24 in OC samples subjected to EIT compared to the control group (Figure 4). Specifically, STK24 expression was observed on and around the HCs near the area of insertion of the electrode. There was high expression of STK24 in the basal turn compared to the apical turn in OCs subjected to EIT.

## 4. Discussion

In this study, we employed iTRAQ™ technology to investigate the host molecular pathways involved in cochlear damage following EIT. Our objective was to identify key proteins and pathways involved in CI trauma, particularly in the middle and basal regions of the cochlea directly impacted by EIT.

Recent clinical and histopathological investigations have demonstrated inner ear injury associated with CI in human subjects. A 2025 study involving adult cochlear implant recipients reported measurable vestibular dysfunction in up to 30% of patients shortly after implantation, as determined by video head impulse testing (vHIT) and caloric assessments [55]. These deficits were particularly pronounced in older adults and individuals with preexisting comorbidities [55]. Complementary findings from the Teenager and Young Adults Cochlear Implant (TAYACI) cohort demonstrated that more than 40% of implanted ears exhibited abnormal cervical and ocular–vestibular-evoked myogenic potentials (cVEMP and oVEMP), indicative of sustained saccular and utricular dysfunction following electrode insertion [56]. A recent postmortem histopathological evaluation of a human temporal bone from a cochlear implant recipient demonstrated extensive electrode-associated cochlear pathology [57]. Quantitative assessment revealed a near-total loss of inner hair cells within the apical turn, indicative of advanced sensory epithelial degeneration. Prominent fibrotic tissue proliferation was identified within both the scala tympani and the scala vestibuli along the electrode trajectory. Additionally, areas of neo-ossification were observed, contributing to luminal narrowing and structural distortion. Notably, the round window membrane was obliterated by a dense fibro-osseous reaction, suggesting a sustained inflammatory and tissue-remodeling response [57]. These findings implicate chronic intracochlear inflammatory processes in the progressive loss of residual hearing and may contribute to delayed auditory decline following implantation. Collectively, these clinical data corroborate the spatially resolved molecular disturbances identified in our ex vivo model and highlight its relevance for studying the mechanisms underlying CI-induced injury.

Utilizing immunofluorescence labeling in conjunction with high-resolution confocal microscopy, we identified a spatially progressive decline in hair cell viability, originating in the apical turn and extending through the middle to the basal turn of the cochlea. This viability gradient was absent in untreated control samples, thereby confirming the successful replication of trauma in the experimental model. Notably, the observed pattern of hair cell degeneration closely parallels the distribution and severity of damage typically reported in clinical cases of cochlear implantation, thereby validating the physiological relevance and translational utility of the model [58,59].

After confirming EIT, we utilized iTRAQ to analyze the upregulated proteins in response to EIT in the OC explants. Among the identified overlaps in upregulated proteins, four proteins, namely Glutamate receptor ionotropic, NMDA 1 (GRIN1), Tumor necrosis factor receptor superfamily member 8 (TNFRSF8), Optineurin (OPTN), and Serine/threonine-protein kinase 24 (STK24), displayed high expression levels in the middle and basal turns. While the literature suggests these proteins participate in apoptotic signaling under specific conditions, their roles in cochlear hair cell injury remain to be fully explored. Therefore, we interpret these associations cautiously and recognize that further functional validation is required in the middle and basal turns, corresponding to the areas of direct EIT. GRIN1 is a subunit of the NMDA (N-methyl-D-aspartate) receptor, which is an ionotropic glutamate receptor involved in synaptic plasticity and excitatory neurotransmission. A study by Hardingham et al. observed that although its primary function is synaptic signaling, emerging evidence has found that GRIN1 regulates apoptosis by interacting with various signaling pathways and molecules involved in cell death [60]. TNFRSF8, also known as CD30, is a member of the tumor necrosis factor receptor superfamily. It is primarily known for its role in immune response regulation and cell death signaling [61]. OPTN is a multifunctional protein involved in various cellular processes including cell death regulation. It has been associated with both pro-apoptotic and anti-apoptotic functions, depending on the cellular context [62].

Out of all these proteins, STK24 emerged as a novel marker identified in this study. This protein acts on both serine and threonine residues and promotes apoptosis in response to stress stimuli and caspase activation [63]. It also serves to mediate oxidative-stress-induced cell death by modulating phosphorylation of the JNK pathway, a pathway previously identified by our group in sensory hair cell loss [26]. Prior studies on STK24 have provided valuable insights into its role as a pro-apoptotic kinase involved in cellular stress responses and cell death regulation [64,65]. Other studies on STK24 have also demonstrated its ability to induce tumorigenesis by regulating the STAT3/VEGFA signaling pathway. High levels of STK24 have been associated with a shorter overall survival time among non-small cell lung and breast cancer patients [66]. However, we recognize that this oncologic context may not directly translate to inner ear pathology and should be considered tissue- and context-specific.

To better understand the high basal and middle turn expression of STK24 indicated by iTRAQ™ analysis, we performed STK24 immunostaining using confocal microscopy on OC explants. The results confirmed substantial STK24 expression throughout the middle turn and particularly in the basal turn, with sensory cells in the latter region showing marked STK24 expression near the site of direct insertion trauma. These findings not only identify potential therapeutic targets for preventing cochlear damage but also highlight the importance of region-specific responses in understanding the pathophysiology of EIT in the cochlea.

This study has some limitations that should be acknowledged. One limitation is its exclusive focus on upregulated proteins. While this targeted approach allowed for a detailed investigation of the pathways enriched in response to EIT, it may have overlooked biologically relevant changes associated with protein downregulation. A comprehensive analysis incorporating both up- and downregulated proteins may provide a more complete understanding of the proteomic landscape and should be considered in future studies. Although STK24 was validated using immunostaining, additional proteins such as GRIN1 and OPTN were not. This was primarily due to limited tissue availability and the exploratory scope of the current study. Future investigations should incorporate broader protein validation to substantiate these findings. Additionally, while the ex vivo model using neonatal rat OC explants offers a valuable platform for mechanistic exploration, we acknowledge the anatomical and developmental differences compared to the adult human cochlea. These include variations in cochlear maturation, tonotopic organization, and immune responses, which may influence the generalizability of the results. Nevertheless, this model has been extensively validated for studying early cochlear responses and continues to serve as a practical surrogate for evaluating implantation trauma [27,67].

Overall, this study highlights the complexity of the molecular responses initiated by EIT within the cochlea. Through quantitative proteomic analysis, we identified distinct alterations in protein expression associated with trauma-induced cellular stress, particularly in regions most susceptible to mechanical damage. The identification of upregulated apoptotic markers reinforces the hypothesis that sensory cell loss is driven by region-specific activation of pro-death signaling cascades. These findings support a broader framework in which inner ear trauma elicits spatially organized molecular responses, reflecting the underlying topography of cochlear vulnerability. This study contributes to a foundational understanding of the biological processes underlying HP loss following cochlear implantation and sets the stage for future investigations into targeted strategies to mitigate trauma-induced degeneration.

## 5. Conclusions

iTRAQ™ proteomic analysis is a novel approach to identify the effectors of sensory cell damage in response to EIT. This study identified unique proteins involved in EIT-induced sensory cell damage such as STK24. Confocal microscopy confirmed the expression of STK24 in primary OC explants. The clinical implication of this study lies in the potential therapeutic application of inhibitors targeting the identified upregulated proteins, particularly STK24, as novel agents to mitigate EIT. The availability of novel interventions may enhance HP and optimize CI outcomes, ultimately contributing to improved auditory function and quality of life for implanted individuals and their families.

## Figures and Tables

**Table 1:** The upregulated proteins in response to electrode insertion trauma in the organ of Corti explants. The numbers represent fold-change expression levels post-EIT relative to the controls, stratified by cochlear turn. Higher values indicate greater relative upregulation.

Gene	Basal Turn	Middle Turn	Apical Turn	Protein Name
*Actn4*	3 . 436	1 . 08	1.853	Alpha-actinin-4
*Stk24*	3.122	5.643	1 . 393	Serine/threonine-protein kinase 24
*Ctnnb1*	2 . 999	1.767	0.841	Catenin beta-1
*Rbm10*	2 . 468	1.218	2 . 112	RNA-binding protein 10
*Cacna1a*	2 . 339	1.607	0 . 933	Voltage-dependent P/Q-type calcium channel subunit alpha-1A
*Pcsk9*	2.242	0.763	2 . 34	Proprotein convertase subtilisin/kexin type 9
*Grik2*	2 . 112	1.105	1 .8 67	Glutamate receptor ionotropic, kainate 2
*Jmjd6*	2 . 109	1.391	1.667	Bifunctional arginine demethylase and lysyl-hydroxylase JMJD6
*Tnfrsf*	1 . 936	2 . 108	0.722	Tumor necrosis factor receptor superfamily member 8
*Grin1*	1.782	2 . 681	0 . 914	Glutamate receptor ionotropic, NMDA 1
*Tp63*	1.712	1.662	0.709	Tumor protein 63
*Slk*	1.67	0 . 985	0.852	STE20-like serine/threonine-protein kinase
*Optn*	1.653	1.995	0.876	Optineurin
*Bmp2*	1.628	1 . 727	0.76	Bone morphogenetic protein 2
*Slc9a1*	1.49	1.388	0.708	Sodium/hydrogen exchanger 1
*Pkn2*	1.478	1.582	0 . 853	Serine/threonine-protein kinase N2
*Ercc3*	1.445	0.52	1.056	General transcription and DNA repair factor IIH helicase subunit XPB
*Oma1*	1.36	1.402	0 . 997	Metalloendopeptidase OMA1, mitochondrial
*Cast*	1.153	1.116	1.4315	Calpastatin
*Flcn*	1 . 145	1 . 08	0.991	Folliculin
*Atp6v0c*	0.778	0.527	2 . 7	V-type proton ATPase 16 kDa proteolipid subunit
*Ifng*	0.459	1.647	5.096	Interferon gamma
*Aktip*	0 . 269	5.29	7.586	AKT-interacting protein

**Key:**
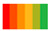
 High to low protein expression.

## Data Availability

Detailed data will be available upon request to the corresponding author.

## Supplementary Materials

The following supporting information can be downloaded at: https://www.mdpi.com/article/10.3390/jcm14145115/s1, Table S1: A summary of experimental design.
