# Supplementary material for: Exploring the Proteomic Landscape of Cochlear Implant Trauma: An iTRAQ-Based Quantitative Analysis Utilizing an Ex Vivo Model
Source: J Clin Med. 2025 Jul 18;14(14):5115. doi: 10.3390/jcm14145115 (PMC12295403; doi:10.3390/jcm14145115)
Supplement: Supplementary file 1 [file jcm-14-05115-s001.zip › jcm-3668170-supplementary.pdf]

Supplementary Table 1: A summary of experimental design

Experimental Group	Number of Animals	Total OC Explants (2 per animal)	Replicates per Condition	OC Explants per Replicate
EIT	36	72	3	24
Control	36	72	3	24
